# Effects of Wildfire Smoke on Volatile Organic Compound (VOC) and PM2.5 Composition in a United States Intermountain Western Valley and Estimation of Human Health Risk

**DOI:** 10.3390/atmos15101172

**Published:** 2024-09-30

**Authors:** Damien T. Ketcherside, Dylan D. Miller, Dalynn R. Kenerson, Phillip S. Scott, John P. Andrew, Melanie A.Y. Bakker, Brandi A. Bundy, Brian K. Grimm, Jiahong Li, Laurel A. Nuñez, Dorian L. Pittman, Reece P. Uhlorn, Nancy A.C. Johnston

**Affiliations:** 1 Lewis-Clark State College, Physical, Life, Movement & Sport Sciences Division, Lewiston, ID 83501 USA;; 2 University of Montana, Department of Chemistry, Missoula, Montana 59812 USA,; 3 University of Washington School of Medicine, Seattle, WA 98195 USA,; 4 Western University of Health Sciences, School of Osteopathic Medicine of the Pacific Northwest, Lebanon, Oregon 97355 USA,; 5 University of Texas Health Science Center, San Antonio, Texas 78229 USA,

**Keywords:** Benzene, PM_2.5_, TD-GC-MS, Northwest, Idaho, wildfire, smoke, health risk

## Abstract

With warmer and drier climate there has been an increase in wildfire events in the Northwest US, posing a potential health risk to downwind communities. The Lewis-Clark Valley (LCV), a small metropolitan area on the Washington/Idaho border in the United States Intermountain West region, was studied over the time period of 2017–2018. The main objective was to determine the community’s exposure to particulate matter (PM2.5) and volatile organic compounds (VOCs) during wildfire smoke events and estimate the associated health risk. VOCs were analyzed in the LCV using sorbent tube sampling and thermal desorption-gas chromatography-mass spectrometry (TD-GC-MS) during several local smoke events in the 2017–2018 fire seasons. PM_2.5_ measurements were obtained from nearby agency monitors. PM_2.5_ reached up to 200 μg/m^3^ in 2017 and over 100 μg/m^3^ in 2018 in the LCV and was observed to be increasing at a rate of 0.10 μg/m^3^/yr over the past two decades. Benzene, a carcinogen and air toxic, was measured with concentrations up to 11 μg/m^3^, over ten times the normal level in some instances in the LCV. The health risk in the LCV from benzene was calculated at 7 extra cancers per million for lifetime exposure and 13 extra cancers per million considering all air toxics measured. The other cities monitored showed similar lifetime cancer risk due to benzene of about 6–7 extra cancers per million. This work is important as it measures ground-level exposures of VOCs and demonstrates decreases in PM air quality over time in the region.

## 1. Introduction

Wildfires have become increasingly prevalent in the Western United States due to climate change, prolonged droughts, and land-use practices [1–4]. During 1984–2011, large US wildfires increased at the rate of seven fires per year, or 355 square kilometers per year [5]. In the Western US, from 1950 until 2019, wildfire frequency and annual area burned followed an exponential growth, and mega-fires have doubled [6]. The forecast for large, simultaneous fires in the west is also increasing [7]. In the Western United States, the number of rain-wetting days between May and September from 1984 to 2015 exhibited a decreasing trend, which has been directly correlated to an increase in forested area burned [8]. Anthropogenic climate impacts have led to an excess of 4.2 million hectares of additional area burned within Western United States forests [9].

As the frequency and area of wildfires escalate, their associated emissions have surged to the forefront of environmental and public health concerns. Biomass burning (BB) contributes to the majority of carbonaceous fine aerosol emissions and is the second largest source of trace gases in the atmosphere [10]. These emissions lead to formation of secondary organic aerosols as well as downwind ozone formation [11]. Studies have shown high concentrations of various VOCs, including alkenes, benzene, furans, phenols, and sulfur containing compounds in wildfire smoke [12–16]. Fine particulate matter (PM_2.5_) has been shown to be increasing in the Northwest US, at a rate of 0.21 (+/− 0.12) μg/m^3^/yr while it is on a downward trend for the rest of the country [17]. Most data to date shows effects from particulates and smoke but overlooks ground level speciated gas components. These can be just as important in human health.

Both the primary and secondary emissions have been shown to be harmful to human health. Exposure to benzene, a trace gas emitted through biomass burning, can lead to acute lymphoblastic leukemia and chronic myeloid leukemia, as well as chromosomal damage in humans [18–19]. Exposure to PM_2.5_ can exacerbate pre-existing respiratory illnesses such as asthma and emphysema [20]. These particles are so small that they make their way into the lung tissue eventually leading to alveolar wall corrosion [21]. While this decreases overall pulmonary function, it also leads to the formation of lung cancers and adenocarcinoma [22]. A study conducted by the American Cancer Society found that with each increase of 10 μg/m^3^ in the PM_2.5_ concentration, mortality due to lung cancer increases by 8% [23]. Models predict a 2.8% increase in overall mortality from short term exposures for each additional 10 μg/m^3^ in the PM_2.5_ concentration [24]. Large cities and urban centers can pose a human health risk to their respective populations due to air pollution, but rural and small urban areas have been frequently overlooked with respect to air pollution from trace gases or particulates. These rural or small urban areas, which have less pollution due to number of people and transportation, tend to be impacted during winter months due to biomass burning for heating homes and during agriculture or wildfire smoke events [25]. Although some studies and regional monitoring have been conducted during wildfire seasons [26–28], there is generally a lack of information from these areas, due to minimal or non-existent monitoring.

The Lewis – Clark Valley (LCV) is a small urban area located along the Northern Idaho – Eastern Washington border with a combined population of about fifty thousand. Wildfire smoke can travel short or long distances and remain stagnant in the LCV for several days or weeks. This leads to spikes in PM_2.5_ concentrations, at times raising the air quality index to unhealthy and very hazardous levels as seen in the last decade [28]. While wildfire smoke does have an impact on the area, it is not the lone source of air pollution. There are various industrial sources within the valley which contribute to the local air pollution, including a Kraft process pulp paper mill. The authors have assessed the valley’s emissions and health risk in the past but without particular focus on wildfire smoke impacts [29]. The Nez Perce Tribe conducted a study on various volatile organic compounds such as benzene and formaldehyde [30–31]. Benzene levels in the LCV were found to be greater than the national mean [31] while formaldehyde was found to be decreasing over time [30]. Health risks attributed to the local pollution were seen as low risk ranging from 2–11 extra cancers per million [29]. In these studies, health risk attributed to wildfire smoke in the LCV has not been reported.

This current work aimed to determine the impact that wildfire smoke has on the concentration of volatile organic compounds (VOCs) and PM_2.5_ in the Lewis – Clark Valley and nearby cities of Coeur d’Alene, Boise and Spokane. Specifically, how did two major smoke events in the summers of 2017–2018 affect health risk based on these exposures to the communities? VOC concentrations obtained previously during this time from thermal desorption-gas chromatography-mass spectrometry (TD-GC-MS) by the coauthors were used with PM_2.5_ values to classify wildfire smoke versus normal air quality days. Human health risk due to benzene and other elevated air toxic exposures in wildfire smoke was determined. PM_2.5_ trends over the past 23 years in the LCV were also investigated. This work addresses the impact of ground level VOCs and PM_2.5_ from wildfire smoke on these communities in the Northwest, where smoke influence is growing at the greatest rate [17].

## 2. Materials and Methods

### 2.1 VOC Data

**Figure 1.**
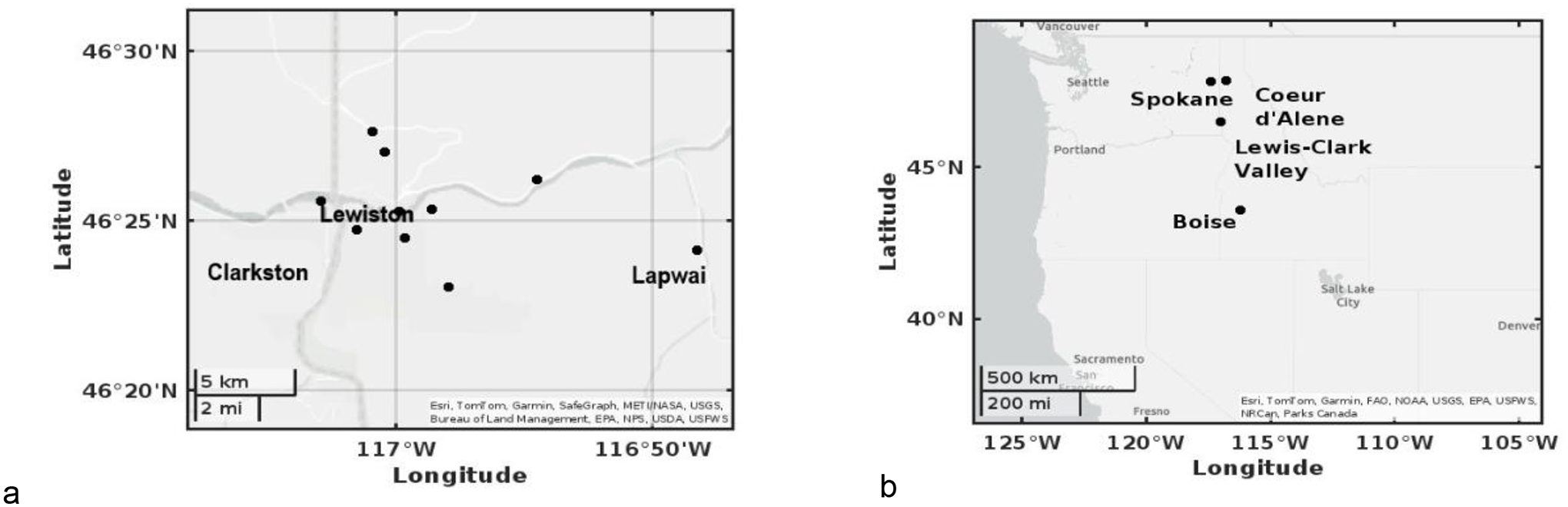
a) Active sampling locations in the LCV b) Passive sampling comparison (biweekly-monthly) at four locations in Idaho and Washington.

VOC concentration data used in this study was obtained previously by the coauthors [29]. In brief, Markes dual sorbent tubes (Tenax^®^TA-Sulficarb) were used for weekly active sampling at 10 sites in the LCV in 2017–2018 (Figure 1a), and Markes Tenax^®^TA sorbent tubes were used for biweekly to monthly passive sampling at LCV and three other cities: Boise, ID, Coeur d’Alene, ID and Spokane, WA in 2018 only (Figure 1b) [29]. Analysis of 50 VOCs using thermal desorption-gas chromatography-mass spectrometry followed US EPA Methods TO-17 and 325 [29, 33, 34]. This dataset of VOCs is available in Scott et al. as supplement materials [29]. For this current study, 337 samples from June – early October 2017–2018 were utilized for the smoke and health risk analysis.

### 2.2 PM_2.5_ Data

Daily mean particulate matter data (PM_2.5_) was obtained from the monitoring sites of the Idaho Department of Environmental Quality and/or Washington Department of Ecology through the US EPA Air Quality System [28]. The monitoring sites were matched to the closest VOC sites and are as follows: Lewiston - Sunset Park, Boise - St. Luke’s in Meridian, Coeur d’Alene - Lancaster Road, and Spokane - Augusta Ave (Figure 1b) [28]. PM_2.5_ data were taken the same day and same hour for comparison with active VOC samples from 2017–2018. Passive samples of VOCs were biweekly to monthly durations and were compared to daily mean PM_2.5_ levels averaged during the respective time period. In addition, the daily mean PM_2.5_ record from 2000–2023 was utilized to observe longer term trends in the LCV [28].

Direct observation of wildfire smoke during grab sampling as well as observation of PM_2.5_ levels were used to designate smoke impacted samples. For the background observations designation, daily mean PM_2.5_ was below 25 μg/m^3^, while biomass burning levels were considered above this threshold during the summer/early fall months (June-October), unless smoke was directly observed. For reference, the regulatory daily 24-hr PM_2.5_ standard is 35 μg/m^3^, and the annual is 9 μg/m^3^ [37]. Student t-test of differences of standard deviation and means (95% confidence level) were performed on VOCs that had at least 20% detection, to determine differences in background versus smoke samples. Correlations between PM_2.5_ and VOCs were also calculated with both the background (BG) and smoke samples (BB) and tested for significance (95% confidence level) using the critical values for the correlation coefficients.

### 2.3 Health Risk Calculations

The effect of exposure to VOCs was determined by use of US EPA exposure methods [38]. This approach uses the mean (or upper confidence level, UCL) exposure concentration multiplied by a risk factor for selected compounds. The unit risk factors and risk categories are available through Integrated Risk Information System [39]. The following equations were used in calculating risk [38]:

(Equation 1) Risk=EC×IUR

where EC is the exposure concentration in μg/m^3^, calculated by Equation 2,

(Equation 2) $$EC=\frac{\sum (CA\times ET\times EF\times ED)}{AT}$$

and IUR is the inhalation unit risk in (μg/m^3^)^−1^, or the lifetime risk of developing cancer for an individual exposed to 1 μg/m^3^ of the chemical. CA is the measured concentration in μg/m^3^, which was taken as the UCL or the upper confidence limit (95%) of the chemical, ET is the exposure time (hrs/day), or 24 hrs/day (residential), EF is the exposure frequency (days/year), 30 days for smoke and 320 days for background, totaling 350/year (residential), ED is the exposure duration (years), 26 years (residential) or 70 years (lifetime), AT is the averaging time (hrs) = 70 years × 365 days/year × 24 hrs/day = 6 × 10^5^ hrs (expected lifetime) [39]. For benzene, Risk Factor = EC (μg/m^3^) × (7.8 × 10^−6^ m^3^/μg), where the upper estimate of IUR is used. [40]

For non-carcinogenic VOCs, a hazard quotient (HQ) was calculated based on the inhalation chronic reference concentration (RfC) in (μg/m^3^), where EC was calculated using ET of 24 hrs, ED of 30 days for smoke and 335 days for background, and ED of 26 and 70 years for residential and lifetime exposures [38]:

(Equation 3) $$HQ=\frac{EC}{RfC}$$

Specifically, the HQ was used to assess the non-carcinogenic effects from compounds like benzene and toluene. An HQ greater than one indicates that estimated exposure may cause non-carcinogenic health effects [38]. If the HQ is less than one, then non-carcinogenic health effects should not be likely to occur. The hazard index (HI) is the sum of HQs of each compound [38].

## 3. Results/Discussion

### 3.1 Observed Smoke Events and PM 2.5 Trends

**Figure 2.**
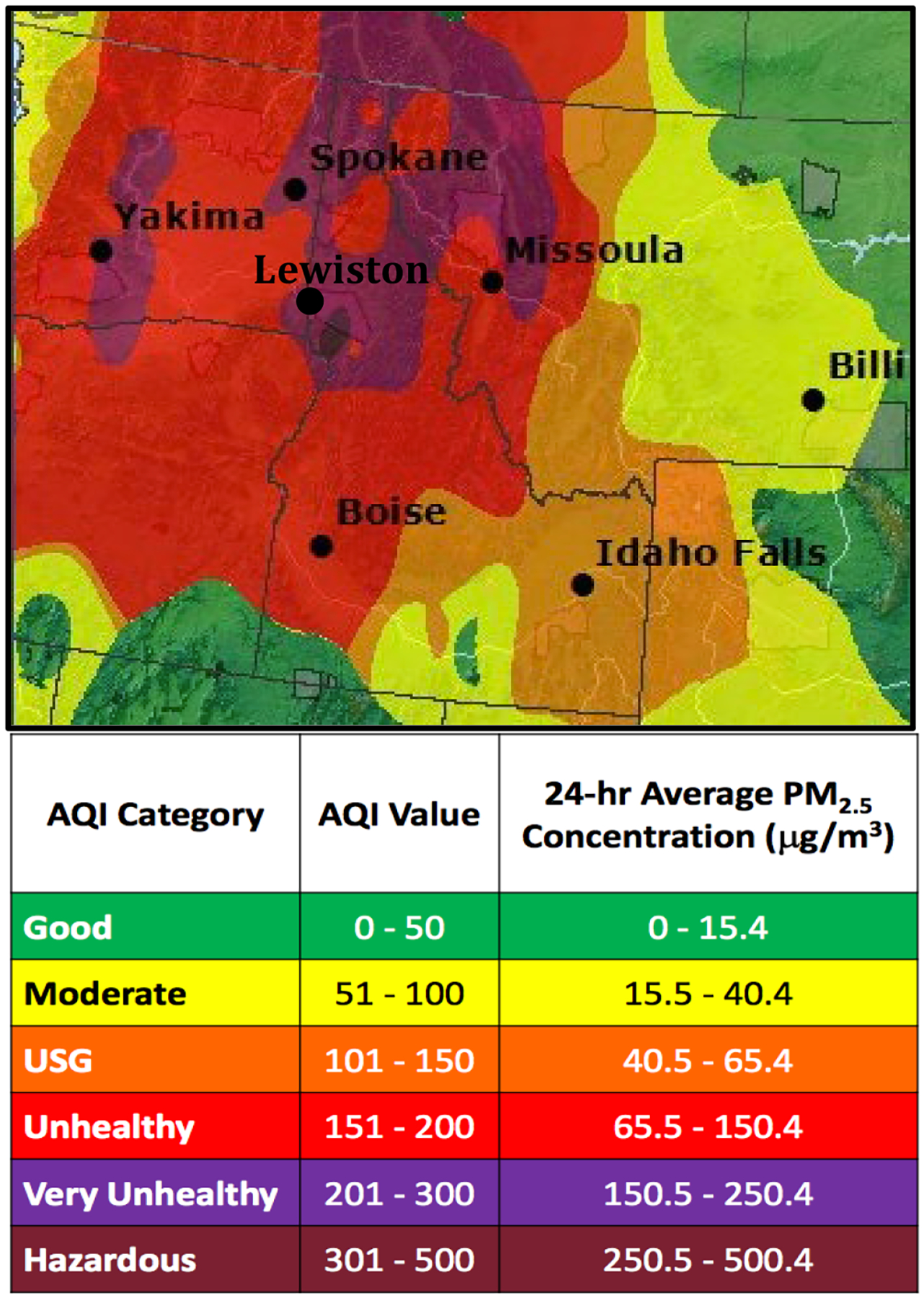
PM_2.5_ AQI in the Northwest US during 7-September 2018 smoke event [41]. The legend shows the concentrations for various levels, where USG refers to unhealthy for sensitive groups. The LCV and other sites sampled were in the unhealthy (red) to very unhealthy (purple) range on this day.

**Figure 3.**
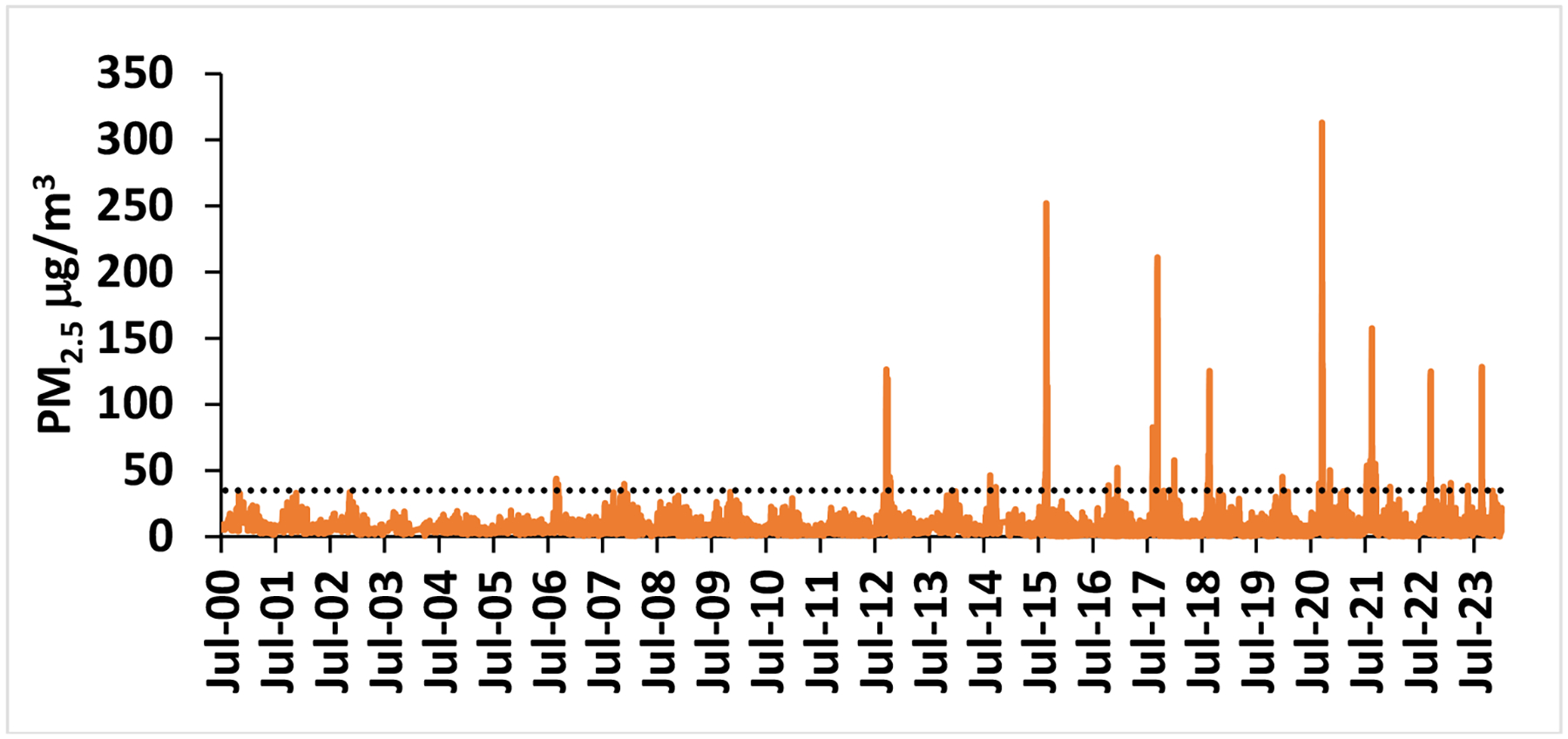
PM_2.5_ (μg/m^3^) in the LCV from July 2000 through December 2023 [28]. The dashed horizontal line is the US EPA 24-hr average air quality standard for PM_2.5_, 35 μg/m^3^ [37].

Both in the summers of 2017 and 2018, smoke events were observed in the LCV. Air quality was poor, and at times was at the very unhealthy to hazardous level. One example is shown on 7-September 2017 in Figure 2, when a severe biomass burning event was taking place in the Northwest US. During the two summer seasons of 2017–2018, 245 background samples and 92 smoke samples were taken in the LCV. Figure 3 demonstrates PM_2.5_ changes over the past 23 years in the LCV [28]. The horizontal line is the daily air quality standard of 35 μg/m^3^ [37]. There is an increasing trend with a slope of 0.10 μg/m^3^/yr during this time. This value is comparable to the 0.21 μg/m^3^/yr McClure and Jaffe reported for the Northwest [17]. From 2012 to 2023, there were 8 years that had summer spikes of PM_2.5_ > 35 μg/m^3^, compared to 2000–2011, where only 2006 had a small spike above this level.

### 3.2 Concentrations of VOCs and PM_2.5_ in Wildfire Smoke in LCV

The most concentrated and/or most detected VOCs of those measured during the June-October periods are shown in Table 1. Benzene, toluene, ethylbenzene and xylenes (BTEX) were all elevated in smoke compared to the background samples with 2017 means of (2.14, 2.98, 0.42, 1.50 μg/m^3^) and 2018 means of (1.42, 1.51, 0.21, 0.54 μg/m^3^), respectively (Table 1). Other elevated hydrocarbons included naphthalene, p-cymene, and phenol (Table 1).

**Figure 4.**
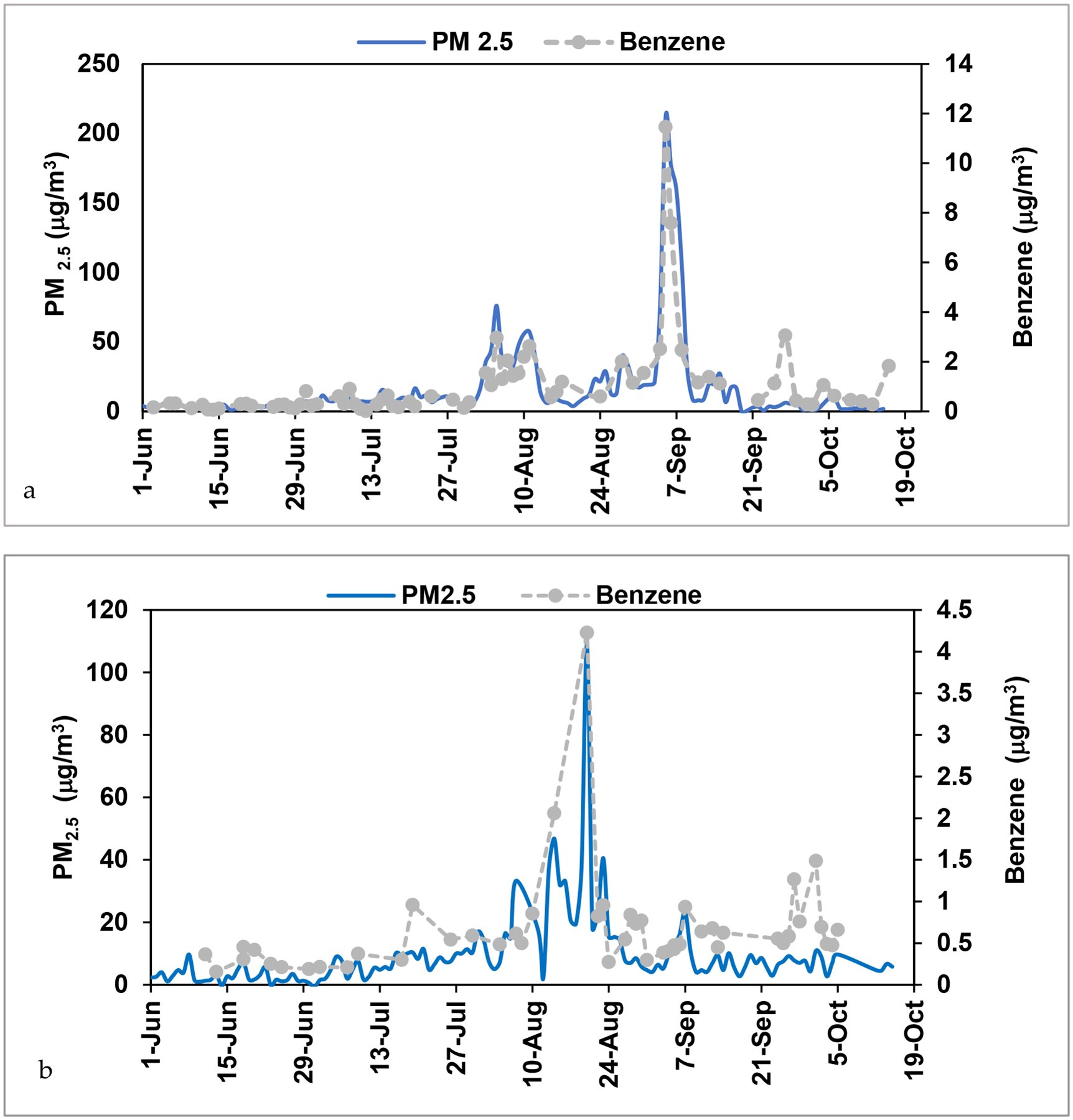
PM_2.5_ and Benzene concentrations in LCV from June to October (a 2017, b 2018). PM_2.5_ daily mean is shown, while benzene was measured weekly and shown with the marker.

**Figure 5.**
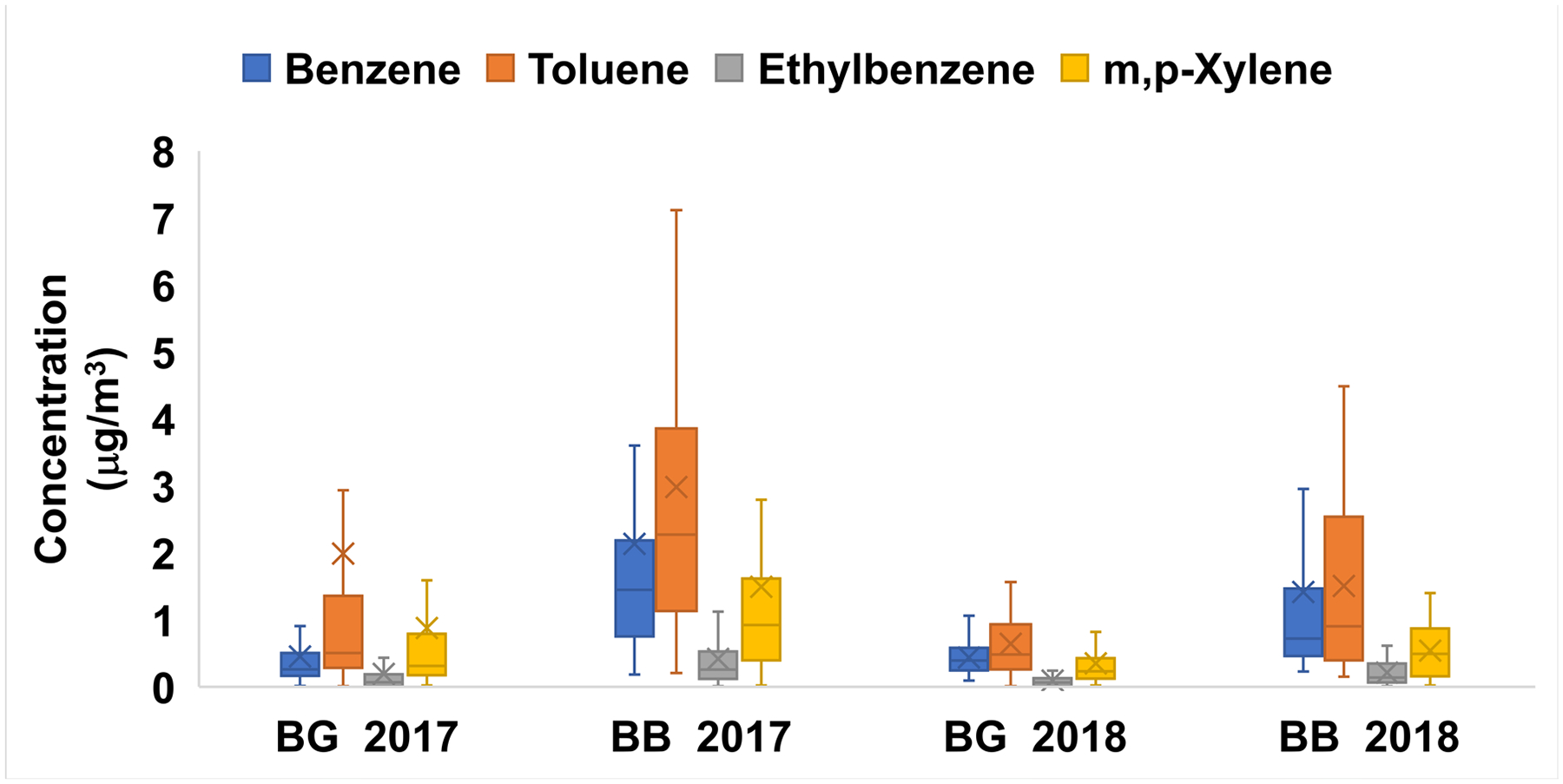
Box and whisker plots of Benzene, Toluene, Ethylbenzene and m,p-Xylene (BTEX) concentrations in LCV for background (BG) and biomass burning (BB) samples collected during from June to October 2017–2018. All BTEX means were significantly different in BB compared to BG except toluene in 2017.

Although halogenated air toxics were measured, these were not statistically increased in wildfire smoke and/or were not believed to come from wildfire sources as they are anthropogenic. 2017 means of VOCs in smoke samples were higher compared to 2018, and this was also seen in PM_2.5_ levels, indicating a more severe fire season in the LCV (Table 1, Figure 4). Benzene time series were plotted with PM_2.5_ for each year in Figure 4. Two large wildfire smoke events as evidence by the maximum benzene and PM_2.5_ levels in (Figure 4) peaked on 5-September 2017 (11.46 μg/m^3^ benzene, 211 μg/m^3^ PM_2.5_) and 20-August 2018 (4.22 μg/m^3^ benzene, 109 μg/m^3^ PM_2.5_). Benzene and PM_2.5_ behaved very similarly to each other during the biomass burning smoke episodes. This relationship supports that the benzene increases were a result of the wildfire smoke. BTEX distributions for background and wildfire smoke or biomass burning samples are shown in Figure 5. All species were elevated in smoke samples with 2017 showing higher means than 2018, and all the differences from background were statistically significant except toluene in 2017. There were also local emissions of benzene (and BTEX) including automobiles and industry, but there was a five-fold increase in the 2017 mean values of benzene in smoke (biomass burning) versus background samples (Table 1). Other sources of VOCs in the LCV were appointed to be the paper mill, secondary formation, traffic and biogenic sources and will not be an in-depth anlaysis here [29–30].

**Figure 6.**
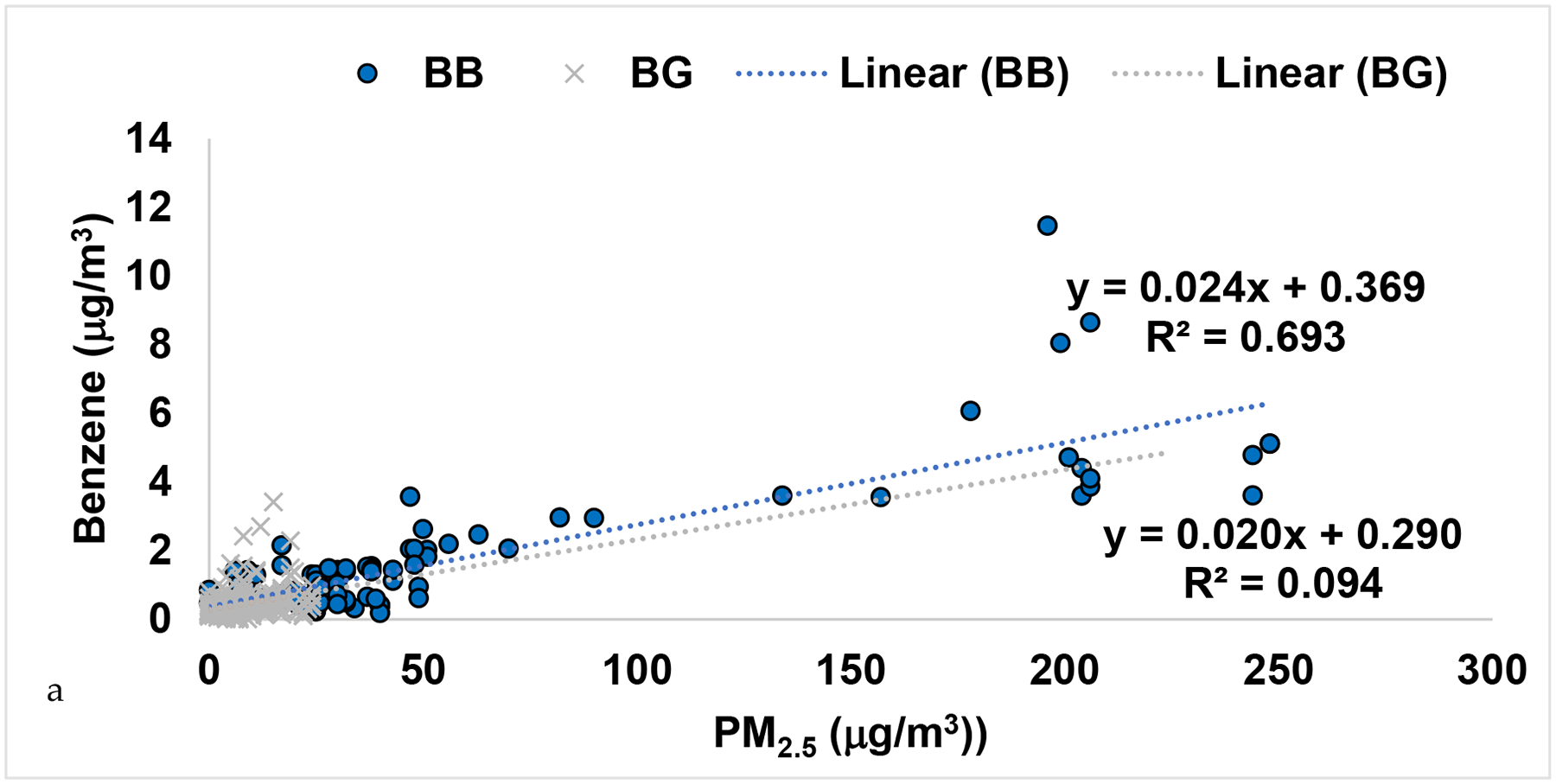

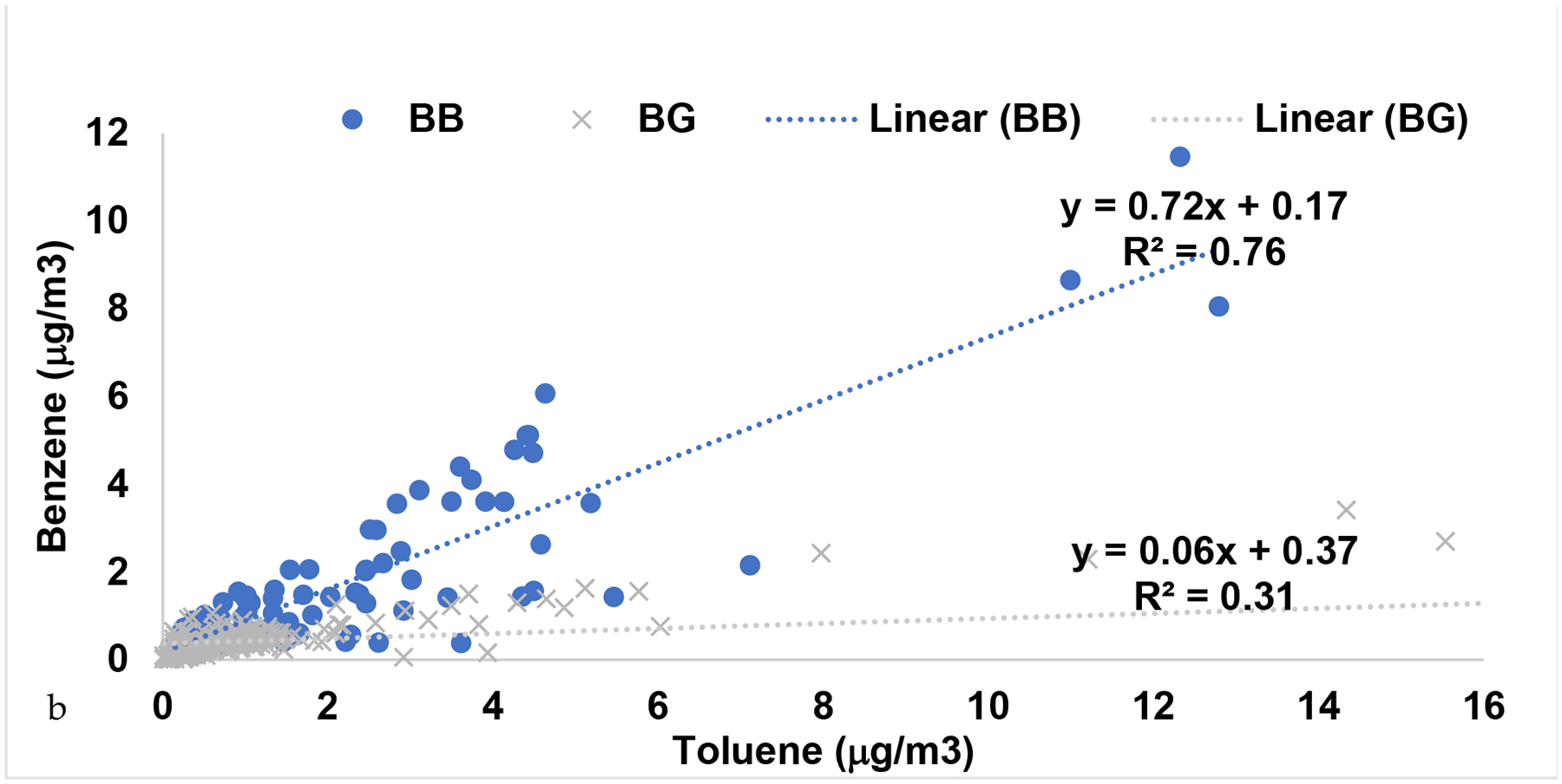
Correlation plots including both 2017–2018 samples in the LCV of a) Benzene and PM_2.5_ and b) Benzene and Toluene, in units of μg/m^3^.

Several species were correlated with PM_2.5_ in the smoke samples versus the background (Table 2). Benzene and PM_2.5_ were correlated in smoke samples with R^2^ value of 0.69 as shown in Figure 6a and Table 2. In fact, the slope of benzene to PM_2.5_ was 0.024 (μg/m^3^:μg/m^3^) or 0.075 (ppb:μg/m^3^). This was comparable to the ratios of benzene to PM_1_ of 0.092 (ppb:μg/m^3^) found in the WE-CAN airborne study [13]. The non-benzene BTEX compounds did not correlate as well to PM_2.5_ with R^2^ values of 0.39, 0.29, and 0.08 respectively, as they were elevated on both background and smoky days (Table 2). In addition, naphthalene and p-cymene were moderately correlated to PM_2.5_ with R^2^ of 0.56 and 0.54 (Table 2). Dimethyl disulfide (DMDS) was not observed in each sample and its R^2^ with PM_2.5_ was low (0.04). Dimethyl sulfide (DMS) was observed at high levels in both background and smoke events due to the location of a local paper mill in Lewiston [29]. DMS did not have a correlation with PM_2.5_ (0), even though it has been shown to be emitted from biomass burning [42]. It is likely that the wildfire smoke was aged and DMS and DMDS decayed in this smoke before it got to the region since the lifetime of these compounds are a day or less.

BTEX species were also correlated to each other in smoke samples. Benzene and toluene correlation is shown in Figure 6b, with R^2^ of 0.76 for smoke samples, and R^2^ reduced to 0.31 for background samples. The slope was higher for smoke (BB) samples as well, showing benzene levels increased in smoke than without. Smoke samples are expected to have a higher benzene/toluene (B/T) ratio compared to traffic emissions, and aged samples from either source should also have higher B/T ratios compared to samples closer to source, as toluene will react in the atmosphere more quickly with OH radicals, one of the main sinks [43]. The B/T (mole/mole) ratio was on average 1.18 +/−0.70 (range 0.12–3.27) in BB samples, and 0.94 +/− 1.02 (range 0.02–11.04) in BG samples. These values are consistent with other studies reporting B/T ratios greater than one for smoke samples [44–46] and less than one for traffic [45].

### 3.3 Passive Sampling Comparison Between Cities

**Figure 7.**
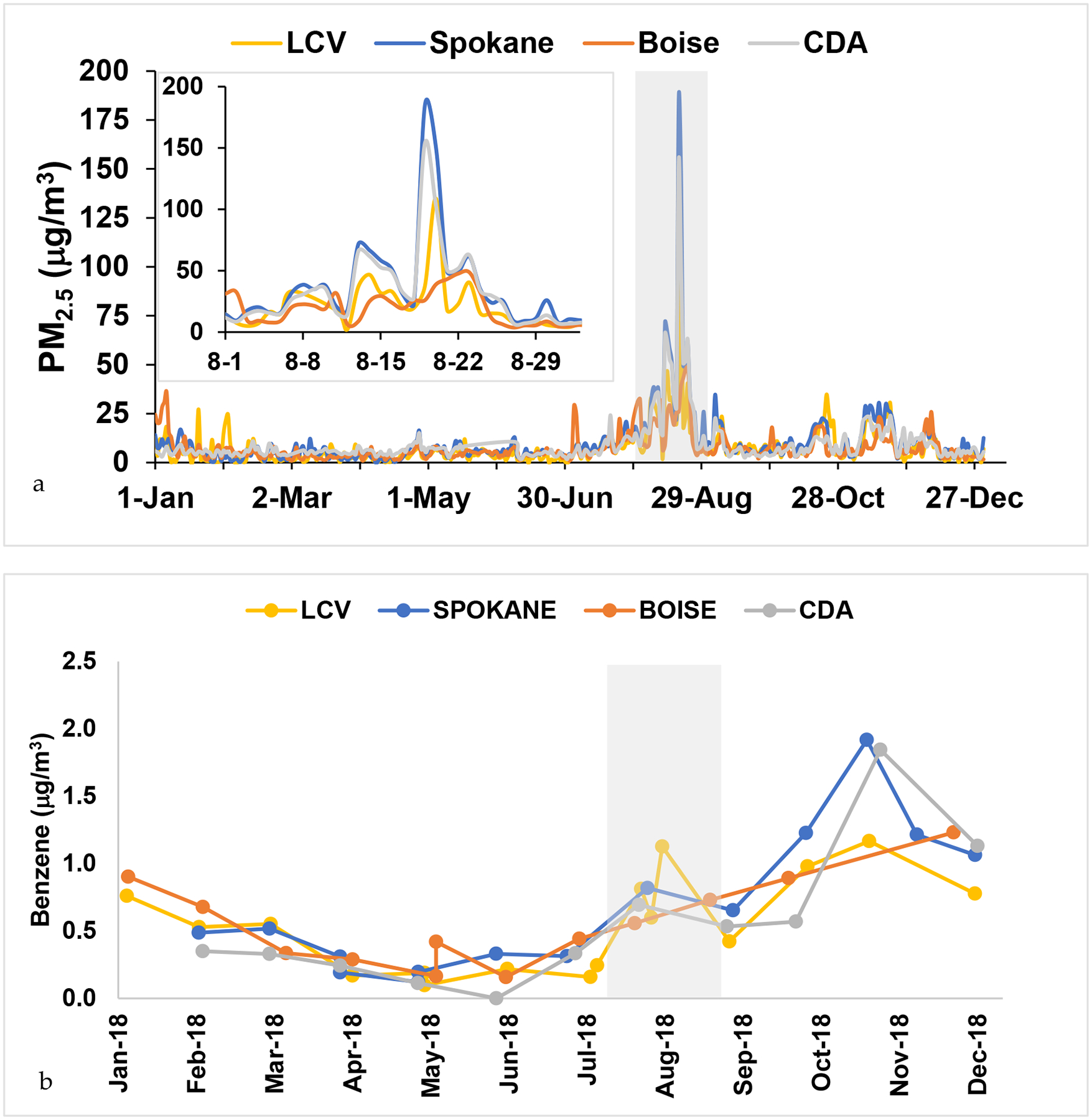
a) PM_2.5_ timeline for 2018 in LCV and three comparison cities, Spokane, Boise, and Coeur d’Alene (CDA) with insert zooming in on the smoke event (shaded gray) in August. b) Benzene averaged from passive sampling at four locations in 2018, with August smoke event shaded in gray.

In order to better understand the long-term exposure of these compounds (versus a compilation of grab samples), passive samplers were used in 4 locations with a sample taken for 2–4 weeks (Figure 1b). Only benzene, toluene and xylene were examined, as these compounds had available uptake rates on the Tenax^®^TA sorbent [36]. These results are shown in Figure 7b. Smoke influence was present in August 2018 at all locations, consistent with the grab sampling results of LCV.

The whole region experienced this smoke event, with highest levels of PM_2.5_ in Spokane (185 μg/m^3^) and Coeur d’Alene (CDA) (154 μg/m^3^) on 19-August 2018. LCV peaked the next day at 109 μg/m^3^ and Boise did not have as much smoke evidenced by PM_2.5_ of 43 μg/m^3^ (Figure 7a). Benzene levels also had a peak in August 2018 coinciding with the smoke event, but had a second and higher peak in November in the northern sites (LCV, Spokane and CDA)(Figure 7b). This could be from the use of wood burning stoves and/or cold weather inversions, as the PM_2.5_ also has a small increase during that time. In general, the Northwest region experienced similar values of PM_2.5_ and benzene levels ranged from 0.2–1.1 μg/m^3^, which were slightly lower than the active samples (Figure 7b). This was due to the passive monthly averaging versus the short-term active sampling.

### 3.4 Health Risk Assessment

Of the compounds measured, benzene was the main health risk (due to it being a carcinogen) aside from PM_2.5_ in wildfire smoke. The inhalation unit risk (IUR) for benzene of 7.8 × 10^−6^ (μg/m^3^)^−1^ was used in this analysis to err on side of protection [36]. This corresponds to the risk of cancer when exposed to 1 μg/m^3^ of benzene throughout a lifetime. This level of benzene (1 μg/m^3^) is representative in the U.S. with associated health risk of 1–10 × 10^−6^ according to the air toxic inventory and ambient air monitoring [47]. The background mean in this study was 0.26 and 0.30 μg/m^3^ for 2017 and 2018. The biomass burning event grab sampling means for LCV were 2.62 μg/m^3^ and 1.73 μg/m^3^ for 2017 and 2018. The corresponding cancer risk factors were 3 × 10^−6^ and 7 × 10^−6^ for residential and lifetime scenarios for benzene alone, and 5 × 10^−6^ and 13 × 10^−6^ when including trichloromethane (Table 3). Trichloromethane (chloroform) is not associated with wildfires, but the paper mill emissions in this region [29]. Non-cancer risk was calculated for the smoke and background exposures for all VOCs with an available chronic inhalation reference concentration (RfC) [40]. The results were HQ values under 1, with highest values of benzene and naphthalene of about 0.03 each, and HI of 0.08, which is under the value of 1 (Table 3). This suggests there is a low probability of a non-cancer health event occurring. Each VOC has specific target organs and the results may or may not be summative, and this is beyond the scope of our analysis.

When considering instead the monthly passive samples obtained, the benzene range of 0.22–0.56 μg/m^3^ translates to cancer risk of 2 × 10^−6^ and 5 × 10^−6^ for residential and lifetime scenarios in the LCV (Table 4). This was slightly less than the analysis of the grab sampling, and the sampling size was smaller at n=13 (monthly samples). The other areas were about the same, with Spokane having 3 × 10^−6^ and 7 × 10^−6^ cancer risk (residential, lifetime), and Boise and Coeur d’Alene (CDA) both with 2 × 10^−6^ residential cancer risk and 6 × 10^−6^ and 7 × 10^−6^ lifetime cancer risk, respectively (Table 4). The HI was about the same for all of the areas, ranging from 0.02–0.03 for lifetime exposure to benzene.

The health implication found that wildfire smoke events increase the lifetime cancer risk to the community by up to 7 extra cancers per million in the LCV and this is similar to surrounding areas in the Northwest.

Although the health risk due to PM_2.5_ is a bit more complex, a 2.8% increase of mortality for every 10 μg/m^3^ increase in PM_2.5_ was estimated for short-term exposures [24]. For the smoke events observed in this study, increases of up to 200 μg/m^3^ would imply a 56% increase in mortality associated with such events. While this seems large, it warrants epidemiological studies to further study the severity of such events.

### 3.5 Comparison to Previous Works

The authors’ earlier health risk assessment in the LCV showed cancer risk from VOCs to be in the range of 2–11 extra cancers per million [25]. The current analysis confirms that, with 2–13 extra cancers per million using the background and smoke criteria versus aggregating the VOCs without this distinction. Miller et al. [22] studied benzene and other VOCs in 2019 emissions in the LCV as well as Boise and Spokane, with a cancer risk of only 1 extra cancer per million due to benzene. 2019 had much less smoke impact than 2017–2018 studied here. Another study in the region was closer to the wildfires themselves, and this estimated cancer risk to be 1–19 extra cancers per million, due to benzene alone, which again, is comparable [46]. Few other studies have focused on VOCs in wildfire smoke and assessed cancer risk. O’Dell et al. [13] used ratios of benzene and other air toxics with PM_2.5_ at higher altitude, to estimate risk at ground levels, in the range of 2–10 extra cancers per million in Idaho/Eastern Washington during 2018, which is comparable to the present study. Navarro et al. [48] used the benzene to PM_1_ ratios observed by O’Dell et al. [13] to estimate exposure concentrations of fire fighters in the Western US. The benzene levels on the front lines were much higher than in the communities and these were closer to levels observed by Dickinson et al. [46] which were also close to the fires. Jin et al.[16] and Wang et al. [49] observed benzene to be about 1 μg/m^3^ in wildfire smoke. These and others are summarized in Table 5.

### 3.6 Limitations

The lifetime relative risk for increased benzene exposure due to wildfire smoke was elevated for LCV residents. However, acute and sub-chronic problems may occur as a result of wildfire smoke. This analysis does not consider the PM_2.5_ health effects, which can also contribute to illness. In addition, these are calculated risks of cancer and non-cancer events due to increased air toxics during wildfire smoke episodes. There are many other potential causes of cancer, and this study only addresses the air toxics that were measured. Thus, this is likely an underestimate of total health risk via all exposures and mechanisms.

Uncertainties are estimated at 10% for the original VOC measurements, as well as using the upper confidence limit (UCL) in the risk calculations for the exposure concentration calculations. The risk analysis uses a value for IUR that is the upper limit (7.8 × 10^−6^ (μg/m^3^)^−1^, and the lower limit is reported as 2.2 × 10^−6^ (μg/m^3^)^−1^, which is about a third of the value used in this study [40].

## 4. Conclusions

This study focused on the impact of wildfire smoke on VOCs in a small metropolitan, intermountain valley in the Western US and surrounding cities from 2017–2018. Wildfire smoke events occurred in the Lewis-Clark Valley in September 2017 and August 2018 that elevated PM_2.5_ for about 30 days and up to 200 μg/m^3^. Several VOCs were elevated in smoke including air toxics benzene, toluene, ethylbenzene, xylenes, naphthalene, and p-cymene. PM_2.5_ increased in the LCV by 0.10 μg/m^3^/yr over the past 23 years, showing the impact of wildfires on the region. Although the lifetime hazard index was below one and considered low risk, there was an associated 7 extra cancers per million for lifetime exposure. This extra risk was consistent at Boise, Coeur d’Alene and Spokane (6–7 extra cancers per million) due to lifetime benzene exposure from smoke. This work is important as it assesses exposure at the ground level, where communities are exposed, in the Northwest US region which is plagued by wildfire smoke events.

## Figures and Tables

**Table 1:** 2017–2018 statistics divided by year, background (BG) and smoke or biomass burning (BB), as well as compounds with statistically significant increases from BG to BB (in bold). All concentrations are in μg/m^3^.

2017	Background samples (n=125)				Biomass Burning samples (n=44)			
Compound	% ND	Min - Max	Mean ± S	UCL	% ND	Min - Max	Mean ± S	UCL
**Benzene**	5	0.02 – 3.42	0.45 ± 0.54	0.67	0	0.18 – 11.46	2.14 ± 2.30	2.89
**Benzene, propyl-**	75	0.02 – 0.61	0.05 ± 0.10	0.09	39	0.02 – 0.56	0.10 ± 0.12	0.18
**Dichlorodifluoromethane**	0	2.51 – 17.87	6.31 ± 2.91	6.75	0	5.49 – 12.90	8.71 ± 1.32	9.04
**Ethylbenzene**	32	0.01 – 2.55	0.20 ± 0.38	0.34	5	0.01 – 1.78	0.42 ± 0.44	0.71
**m,p-Xylene**	10	0.02 – 12.31	0.88 ± 1.71	1.55	2	0.02 – 6.25	1.50 ± 1.60	2.50
**Naphthalene**	79	0.01 – 0.89	0.05 ± 0.12	0.08	59	0.01 – 0.89	0.12 ± 0.18	0.18
**p-cymene**	61	0.01 – 1.24	0.14 ± 0.25	0.19	7	0.01 – 2.57	0.49 ± 0.49	0.65
**Phenol**	63	0.02 – 4.58	0.26 ± 0.62	0.32	2	0.02 – 2.37	0.66 ± 0.43	0.79
**Trichloromonofluoromethane**	1	0.01 – 4.73	0.97 ± 0.62	1.07	0	0.37 – 2.69	1.21 ± 0.56	1.35
Benzene, 1,2,4 trimethyl	85	0.06 – 4.16	0.22 ± 0.57	0.34	70	0.06 – 0.74	0.13 ± 0.14	0.17
Dimethyl sulfide	50	0.02 – 40.55	1.99 ± 5.80	3.97	41	0.02 – 54.99	3.28 ± 8.77	7.49
Disulfide, dimethyl	78	0.01 – 25.57	0.56 ± 3.12	1.80	68	0.01 – 5.64	0.27 ± 0.87	0.75
Mesitylene	67	0.02 – 1.15	0.08 ± 0.17	0.15	50	0.02 – 0.73	0.11 ± 0.15	0.16
Methylene chloride	6	0.02 – 2.32	0.17 ± 0.21	0.19	0	0.08 – 1.71	0.23 ± 0.24	0.29
Tetrachloroethylene	74	0.01 – 1.85	0.07 ± 0.23	0.16	45	0.01 – 0.62	0.10 ± 0.14	0.19
Toluene	1	0.01 – 41.95	1.99 ± 5.57	4.17	0	0.21 – 12.8	2.98 ± 2.92	3.75
Trichloromethane	29	0.01 – 1.55	0.14 ± 0.22	0.23	20	0.01 – 1.13	0.21 ± 0.23	0.29
2018	Background samples (n=120)				Biomass Burning samples (n=48)			
Compound	% ND	Min - Max	Mean ± S	UCL	% ND	Min - Max	Mean ± S	UCL
**Benzene**	0	0.09 – 1.26	0.44 ± 0.23	0.48	0	0.23 – 5.12	1.42 ± 1.53	2.38
**Ethylbenzene**	42	0.01 – 1.14	0.09 ± 0.14	0.14	19	0.01 – 0.62	0.21 ± 0.20	0.27
**m,p-Xylene**	22	0.02 – 5.09	0.35 ± 0.58	0.55	12	0.02 – 2.32	0.54 ± 0.47	0.69
**Naphthalene**	78	0.01 – 0.36	0.03 ± 0.05	0.04	65	0.01 – 0.37	0.08 ± 0.12	0.11
**p-cymene**	40	0.01 – 0.80	0.14 ± 0.18	0.21	21	0.01 – 2.16	0.38 ± 0.48	1.17
**Toluene**	1	0.01 – 3.93	0.65 ± 0.59	0.75	0	0.15 – 4.49	1.51 ± 1.44	2.32
Benzene, propyl-	84	0.02 – 0.31	0.03 ± 0.04	0.05	65	0.02 – 0.14	0.04 ± 0.04	0.05
Carbon Tetrachloride	47	0.03 – 0.50	0.22 ± 0.18	0.25	79	0.03 – 0.46	0.10 ± 0.14	0.13
Dichlorodifluoromethane	0	3.83 – 5.97	4.70 ± 0.38	4.76	0	3.95 – 5.50	4.59 ± 0.40	4.69
Dimethyl sulfide	15	0.02 – 16.00	1.75 ± 3.30	3.07	12	0.02 – 13.88	2.45 ± 3.37	10.33
Disulfide, dimethyl	85	0.01 – 5.25	0.25 ± 0.91	0.10	75	0.01 – 3.34	0.16 ± 0.53	0.43
Methylene chloride	2	0.02 – 0.71	0.20 ± 0.08	0.21	0	0.10 – 0.25	0.18 ± 0.03	0.19
Tetrachloroethylene	72	0.01 – 0.47	0.05 ± 0.08	0.05	71	0.01 – 0.34	0.03 ± 0.06	0.02
Trichloromethane	8	0.01 – 3.33	0.25 ± 0.39	0.40	17	0.01 – 1.07	0.23 ± 0.23	0.48
Trichloromonofluoromethane	0	0.29 – 2.40	1.33 ± 0.40	1.39	0	0.52 – 2.11	1.37 ± 0.35	1.46

**Table 2.** Coefficient of Determination values for VOCs with PM_2.5_ in both background (BG) and biomass burning (BB) samples from 2017–2018. Shaded values were significant at the 95% confidence level and bolded values were strong correlations over 0.5.

	PM_2.5_ BG	PM_2.5_ BB
Benzene	0.09	**0.69**
Benzene, 1,2,4 trimethyl	0.00	0.03
Benzene, propyl-	0.03	0.25
Carbon Tetrachloride	0.03	0.03
Dichlorodifluoromethane	0.01	0.02
Dimethyl sulfide	0.05	0.00
Disulfide, dimethyl	0.00	0.04
Ethylbenzene	0.02	0.29
m,p-Xylene	0.02	0.09
Mesitylene	0.02	0.04
Methylene chloride	0.01	0.00
Naphthalene	0.01	**0.56**
p-cymene	0.11	**0.54**
Phenol	0.00	0.01
Tetrachloroethylene	0.02	0.01
Toluene	0.02	0.39
Trichloromethane	0.13	0.00
Trichloromonofluoromethane	0.01	0.01

**Table 3.** Cancer risk analysis of LCV, including smoke or biomass burning exposures (BB) of 30 days and background (BG), repeating for 26 years (Residential) and 70 years (Lifetime). CA is based on the upper confidence level of concentrations in μg/m^3^ obtained via active sampling. EC is the exposure concentration in μg/m^3^, calculated for both residential (Res) and lifetime (Life) scenarios. The inhalation unit risk (IUR) of 7.8 ×10^−6^ (μg/m^3^)^−1^ and the Reference Concentration (RfC) of 30 μg/m^3^ were used for Benzene Cancer Risk calculations, and Hazard Quotient (HQ), respectively [40].

Compound	CA BG	CA BB	EC BG Res	EC BB Res	EC BG Life	EC BB Life	IUR	RfC	Cancer Risk Res	Cancer Risk Life	HQ
Benzene	0.7	2.941	0.23	0.09	0.64	0.24	7.80E-06	30	2E-06	7E-06	2.9E-02
Chloroform	0.23	0.52	0.07	0.02	0.21	0.04	2.30E-05	98	2E-06	6E-06	2.6E-03
Methylene Chloride	0.22	0.33	0.07	0.01	0.20	0.03	1.00E-08	600	8E-10	2E-09	3.8E-04
Tetrachloroethylene	0.05	0.18	0.02	0.01	0.04	0.01	2.60E-07	40	6E-09	2E-08	1.5E-03
Benzene, 1,2,4 trimethyl	0.33	0.15	0.11	0.00	0.30	0.01	NA	60			5.2E-03
Benzene, propyl	0.07	0.23	0.02	0.01	0.06	0.02	NA	1000			7.9E-05
Ethylbenzene	0.31	0.57	0.10	0.02	0.29	0.05	NA	1000			3.3E-04
Mesitylene	0.11	0.11	0.04	0.00	0.10	0.01	NA	60			1.9E-03
Naphthalene	0.07	0.19	0.02	0.01	0.06	0.02	NA	3			2.5E-02
Toluene	3.34	4.58	1.09	0.14	3.06	0.38	NA	5000			6.9E-04
Xylene (m,p)	1.46	2.61	0.47	0.08	1.34	0.21	NA	100			1.6E-02
Cumulative									5E-06	1.3E-05	8.3E-02

**Table 4.** Comparison of health risk during smoke events for LCV and three cities in 2018, using the upper confidence level of benzene concentrations in μg/m^3^ obtained via passive sampling (CA). BG represents background and BB represents smoke or biomass burning samples. EC is the exposure concentration in μg/m^3^, calculated for both residential (Res) and lifetime (Life) scenarios. The inhalation unit risk (IUR) of 7.8 ×10^−6^ (μg/m^3^)^−1^ and the Reference Concentration (RfC) of 30 μg/m^3^ were used for the Cancer Risk calculations, and Hazard Quotient (HQ), respectively [40].

Site	CA BG	CA BB	EC BG Resident	EC BB Resident	EC BG Lifetime	EC BB Lifetime	Cancer Risk Resident	Cancer Risk Lifetime	HQ
LCV	0.69	1.15	0.23	0.04	0.61	0.09	2E-06	5E-06	0.02
Boise	0.78	0.56	0.25	0.02	0.68	0.05	2E-06	6E-06	0.02
CDA	0.91	0.69	0.30	0.02	0.80	0.06	2E-06	7E-06	0.03
Spokane	0.97	0.82	0.32	0.03	0.85	0.07	3E-06	7E-06	0.03

**Table 5.** Summary of results from biomass burning VOC measurements in the Western US region, including current study.

Authors	Study Area	Main Pollutant	Benzene Levels Measured	Cancer Health Risk (per million)
Current study	Northwest US, LCV (ground level)	BTEX	0.02–11 μg/m^3^ (0.06–3.6 ppb)	2–13
Dickinson et al., 2022 [46]	Northwest (ground level, near fires)	BTEX	0.06–80 μg/m^3^ (0.02–25 ppb)	1–19
O’Dell et al., 2020 [13]	Western US (airborne)	Benzene, Acrolein, Formaldehyde	0.1–10 μg/m^3^ (0.03–3 ppb)	2–10
Navarro et al., 2021 [49]	Western US (ground level estimates)	Benzene, Acrolein, Formaldehyde	4.5–19 μg/m^3^ (1.4–6 ppb)	NA
Wang et al., 2024 [48]	Northern California (ground level)	BTEX	1 +/− 0.2 μg/m^3^ (0.3 +/− 0.06 ppb)	NA
Jin et al., 2023 [16]	Western US (airborne, ground level)	BTEX, Formaldehyde, Acetaldehyde	0.03–0.96 μg/m^3^ (0.01–0.3 ppb)	NA

## Data Availability

The dataset utilized for this study has been previously published: Scott, P. S., Andrew, J. P., Bundy, B. A., Grimm, B. K., Hamann, M. A., Ketcherside, D. T., Li, J., Manangquil, M. Y., Nuñez, L. A., Pittman, D. L., Rivero-Zevallos, A., Uhlorn, R., & Johnston, N. A. C. (2020). Observations of volatile organic and sulfur compounds in ambient air and health risk assessment near a paper mill in rural Idaho, U. S. A. [Supplement 2]. Atmospheric Pollution Research, 11(10), 1870–1881. https://doi.org/10.1016/j.apr.2020.07.014
